# A Bionic “Trojan Horse”-like Nanovesicle Delivery System Hybridized with BCG Cytoplasmic Membrane and Melanoma Cell Membrane for Cancer Immunotherapy

**DOI:** 10.3390/pharmaceutics17040507

**Published:** 2025-04-11

**Authors:** Yuai Xiao, Kexin Chen, Tianchi Hu, Yuchong Wang, Jing Wang, Chuan Lv, Jianguo Xu, Xinyi Zhang, Ang Li, Bingdi Chen, Ji Zhu, Minliang Wu, Chunyu Xue

**Affiliations:** 1 Department of Plastic Surgery, Changhai Hospital, Naval Medical University, 168 Changhai Road, Shanghai 200433, Chinadrzhuji@163.com (J.Z.); 2 The Institute for Biomedical Engineering & Nano Science, School of Medicine, Tongji University, Shanghai 200331, China

**Keywords:** nanovesicles, cancer immunotherapy, BCG, hybrid membrane

## Abstract

**Background**: In recent years, tumor vaccines have demonstrated unexpected success in cancer treatment. However, it still faces several challenges, including insufficient antigen and adjuvant delivery, unsuitable antigen delivery system, and inadequate antigen-presenting cell (APC) maturation. Antigenic adjuvant co-delivery tactics could be one way to enhance APC maturation. **Methods**: Membrane-fused nanovesicles were synthesized by separating melanoma cell membranes from BCG cytoplasmic membranes. Dynamic light scattering and transmission electron microscopy were used for measuring the vesicles’ size and shape. The uptake of vesicles by mouse bone marrow-derived dendritic cells and the activation of DC cells by vesicles were verified in vitro. In order to further confirm the material’s capacity to activate the immune system and its ability to inhibit tumor growth, the activation of DC and T cells in mouse draining lymph nodes and the concentration of anti-tumor cytokines were measured. **Results**: The hybrid vesicles were homogeneous in size and could facilitate phagocytosis by dendritic cells (DCs). They could also effectively activate DCs and T cells in vitro and in vivo, eliciting anti-tumor immunity. Moreover, the vesicles demonstrated satisfying biosafety with no major side effects. **Conclusions**: Motivated by the myth of the Trojan Horse, we created an antigen-adjuvant-integrated nanovesicle that merges the BCG cytomembrane with the tumor cell membrane, which can achieve immune cell stimulation and tumor antigen delivery simultaneously. In conclusion, these findings support the potential application of dual-membrane fusion nanovesicles as tumor vaccines.

## 1. Introduction

Immunotherapy has shown promising results in tumor treatment by using the patient’s own immune system to kill tumor cells and control tumor progression or even cure cancer [1]. Cancer immunotherapies include immune checkpoint blockade therapy, adoptive immunotherapy, tumor vaccines, and virotherapy [2]. Among them, tumor vaccines can activate T cells to produce specific anti-tumor immunity by activating antigen-presenting cells (APCs) and presenting tumor antigens [3,4,5]. It is one of the highly promising therapeutic techniques that can activate specialized anti-tumor immunity by removing the tumorigenic components of tumor cells and preparing the tumor’s antigenic components as a vaccine. However, the design and components of tumor vaccines can lead to significant differences in their effectiveness in cancer treatment and recurrence prevention [6,7]. Studies have shown that adjuvants and antigens must be delivered in combination to effectively activate APCs [8]. Nanomedicines offer unique opportunities to improve the efficacy of these vaccines. However, only a small part of the currently existing nano-vaccines exhibit the capacity of dendritic cells (DCs) maturation and antigen presentation at the same time [9]. To increase the tumor vaccine’s efficacy in tumor therapy, we need to develop a novel design strategy.

Cell membrane-derived vesicles have received considerable interest in recent years. In spite of carrying metabolites, proteins, lipids, and nucleic acids that contribute to the regulation of the tumor microenvironment, they are also excellent biological carriers that have the capacity to load drugs and have structural remodeling capabilities [10,11]. Cell membrane-derived vesicles can be divided into two groups: extracellular vesicles secreted by cells, like exosomes, apoptotic vesicles, and microbubbles, and artificially synthesized nanovesicles with a similar structure and function [12]. In contrast to extracellular vesicles, nanovesicles have similar functions, but they have higher yields. Nanovesicle is a good platform for delivering and can be synthesized by altering the composition of the membrane to produce fusion vesicles with specific functions [13,14].

Inspired by the story of the Battle of Troy, we wondered if we could design a Trojan Horse-like nano-vaccine that could be used to defend against the growth of tumor cells. This vaccine has the camouflaged appearance of a cancer cell but carries a component to activate immune cells. When it reaches the tumor site, it can precisely activate the immune cells in the tumor microenvironment, just like the soldiers hidden inside the Trojan Horse. In our study, we chose the tumor cell membrane as our “Trojan horse” [15]. Previous studies have demonstrated that tumor cell membranes can present tumor antigens to APCs [16]. Furthermore, compared with tumor exosomes, it is a cell-free therapy without the potential tumorigenic tendency of tumor cells [17]. As for the soldier’s choice, we choose the Bacillus Calmette–Guérin (BCG) vaccine, an attenuated live strain of Mycobacterium bovis. It is one of the most widely used vaccines in the world and is now widely used in the treatment of cancers, including prostate cancer and non-muscle invasive bladder cancer [18,19]. Additionally, previous studies have demonstrated that BCG inhibits the growth of melanoma, which makes it an extremely promising bacterium for melanoma immunotherapy [20]. However, the attempts at clinical application have revealed its shortcomings, such as the limited immune activation produced by paraneoplastic injection of BCG, the risk of tuberculosis dissemination, and some unwanted systemic inflammatory reactions with systemic application of BCG [21]. Therefore, we need to improve the form of BCG delivery to enhance its immune activation capacity and biosafety.

**Scheme 1:**
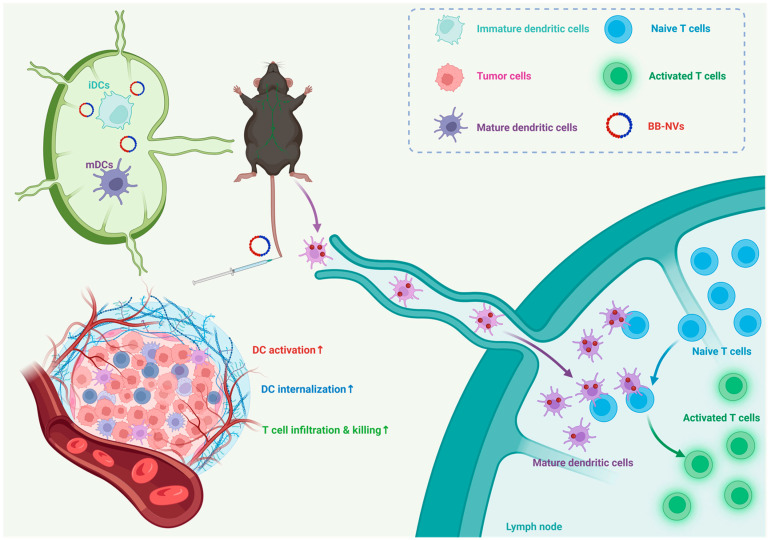
Schematic diagram of the bionic “Trojan horse”-like nanovesicles delivery system.

Thus, in our study, we developed a nano-vaccine with a hybrid membrane, which is the BCG cytoplasmic membrane and the membrane of melanoma cells. The B16 membrane-derived nanovesicles (B16-NVs), BCG-derived nanovesicles (BCG-NVs), and B16-BCG hybrid nanovesicles (BB-NVs) have an exosome-like structure with hydrodynamic sizes of about 200 nm, which can be effectively absorbed by DCs. The BB-NVs can realize the delivery of tumor-specific antigen and adjuvant simultaneously. It contains immunostimulatory molecules on the bacterial membrane that enhance antigen uptake and boost DC maturation, as well as multiple tumor-specific antigens that induce antigen-specific T cell clones. Moreover, we verified that BB-NVs can activate APCs and promote higher levels of anti-tumor cytokine secretion more effectively than B16-NVs and BCG-NVs alone in animal models (Scheme 1).

## 2. Materials and Methods

Material, Cells, and Animals: BCG (R19019) was obtained from Shanghai REBIO (Shanghai, China). A high-pressure extruder (LiposoEasy LE-1 Plus) and polycarbonate porous membranes were purchased from Chuye Bio-Tech (Shanghai, China) Co., Ltd. Dulbecco’s modified Eagle medium (DMEM)/High Glucose, RPMI 1640, phosphate-buffered saline (PBS), and fetal bovine serum (FBS) were purchased from Gibco. Trypsin and penicillin–streptomycin (P/S) were acquired from New Cell & Molecular Biotech Co., Ltd. (Newcastle upon Tyne, UK). The B16-F10 cell line was obtained from the Cell Bank of the Chinese Academy of Sciences, Shanghai, China, and cultured with 10% FBS and 1% P/S at 37 °C and 5% CO_2_. Cells were tested for Mycoplasma, and only Mycoplasma-free cells were used. C57BL/6j female mice aged 5–6 weeks were purchased from JieSiJie Laboratory Animal Co., Ltd. (Shanghai, China) and kept in the specific pathogen-free (SPF) Laboratory Animal Center of Changhai Hospital of Naval Medical University. All animal experimental protocols were approved by the Laboratory Animal Care and Use Committee of the Changhai Hospital of Naval Medical University.

Preparation of BCG membrane nanovesicles: BCG (R19019) was purchased from Shanghai REBIO. BCG was heated in a digital heating circulating water bath at 75 °C for 2 h to eliminate the possible pathogenicity. The BCG freeze-dried powder was dissolved in PBS, and lysozyme was then added to a final concentration of 2 mg/mL. The suspension was incubated at 37 °C for 1 h with shaking at 120 rpm. Then, the bacterial pellets were broken by an ultrasonic reactor at 20 kHz for 10 min and then centrifuged at 12,000 rpm for 5 min to collect the supernatant. The BCG nanovesicles were obtained by using a high-pressure extruder (LiposoEasy LE-1 Plus) to sequentially extrude five times through 10, 5, 1, 0.4, and 0.2 μm polycarbonate membrane filters (Whatman, Maidstone, UK) and then ultracentrifuged at 100,000 g for 2 h at 4 °C to collect the precipitate.

Preparation of tumor cell membrane nanovesicles: The B16-F10 melanoma cancer cells were cultured in a high-glucose DMED medium with 10% FBS and 1% P/S. The cells were collected when they were in the logarithmic growth phase and then resuspended at a concentration of 1.78 × 10^6^ cells/mL in PBS. The pellets were collected and washed with PBS three times. Then, an ultrasonic reactor (20 kHz) was used to break the cells, and then they were centrifuged at 3000 g for 10 min to collect the supernatant. The cell suspension was sequentially extruded five times through 10, 5, 1, 0.4, and 0.2 μm polycarbonate membrane filters (Whatman). Then, the suspension was ultracentrifuged at 100,000× *g* for 2 h at 4 °C to collect the precipitate.

Preparation and characterization of hybrid membrane nanovesicles: Tumor membrane and BCG membrane were sonicated at 125 W, 20 kHz for 5 min and mixed at a mass ratio of 2:1. The suspension was extruded using the same method as described above. DiO and DiI dyes were used to label tumor cell membranes and BCG membranes. Then, confocal microscopy was used to detect the membrane fusion of the hybrid nanovesicles.

In vitro innate immunity experiments: To investigate the capacity of the different membrane nanovesicles in vitro, BMDCs were co-cultured with different nanovesicles. The BMDCs were obtained from the femur and tibia of C57BL/6j female mice aged 4 weeks, and the harvested cells were cultured with RPMI 1640 medium containing 10% FBS, 1% P/S, 20 ng/mL GM-CSF, and 10 ng/mL IL-4. The medium was half changed on day three, and immature BMDCs were collected on day 5. Then, they were co-cultured with different nanovesicles for 24 h. Finally, all cells were centrifuged and collected at 1200 rpm for 5 min and then incubated with 1 μL APC-anti-mouse CD11c, BV421-anti-mouse CD80, and PE-anti-mouse CD86 monoclonal antibody (BD Pharmingen, San Diego, CA, USA). The flow data were analyzed with FlowJoV10 software. The supernatant was collected, and the concentration of proinflammatory cytokines was determined using the mouse TNF-α, IL-6, IL-12 ELISA kit.

In vivo immunogenic effect of the nanovesicles: All C57BL/6j mice were sacrificed on day 14 after the last immunization, and the spleen, tumor tissue, and inguinal lymph nodes were collected. The minced tumor tissues were digested with collagenase (0.5 mg/mL) and DNase (0.1 mg/mL) in RPMI medium with 2% FBS at 37 °C for 1 h. The single-cell suspension of the inguinal lymph nodes and spleen was prepared using a mechanical extrusion method. Then, APC-anti-mouse CD11c, BV421-anti-mouse CD80, and PE-anti-mouse CD86 monoclonal antibodies (BD Pharmingen) were used to measure the maturation of DCs in the inguinal lymph nodes. Additionally, FITC-anti-mouse CD3, PE-anti-mouse CD4, and APC-anti-mouse CD8 were used to detect the activation of T cells in the spleen and tumor tissues. Mouse serum was collected, and the concentration of proinflammatory cytokines was determined using the mouse TNF-α, IL-6, IL-10, and IFN-γ ELISA kit.

In vivo antitumor effect of the hybrid membrane nanovesicles: In order to study the anti-tumor effect, we injected 1 × 10^5^ B16 cells that were suspended in 100 μL PBS on the right wing of the C57BL/6j female mice. Five days later, the tumor volume of the mice reached about 50 mm^3^. Then, the mice were randomly divided into four groups of different treatments (*n* = 5): saline, B16-NV, BCG-NV, and BB-NV. The mice were immunized with the different vaccine formulations (4 mg/kg) on days 1, 7, and 14 after tumor implantation. Tumor volume was calculated according to the following equation: V = (width)^2^ × length/2. All the C57BL/6j mice were sacrificed on day 14 after the last immunization.

Histological evaluation: At the end of the treatment, mice in different groups were euthanized, and the tissues (heart, liver, spleen, lung, kidney, and tumor) were taken out and fixed with 4% paraformaldehyde. The tissues were embedded in paraffin and sectioned at 5 mm intervals for histological evaluation using H&E staining. An optical microscope was used to examine the slides.

Statistical analysis: The experiments were independently repeated three times, and typical data are provided. Statistical analyses were conducted with GraphPad Prism (version 8.0). All of the values are represented as mean ± SD for three biologically separate samples. An unpaired statistical analysis was performed. Two-tailed unpaired Student’s *t*-tests were used to obtain the *p*-values, and the error bars showed the mean ± SEM. * *p* < 0.05, ** *p* < 0.01, *** *p* < 0.001, ns stood for *p* > 0.05.

## 3. Results

### 3.1. Preparation and Characterization of B16-NVs, BCG-NVs, and BB-NVs

**Figure 1:**
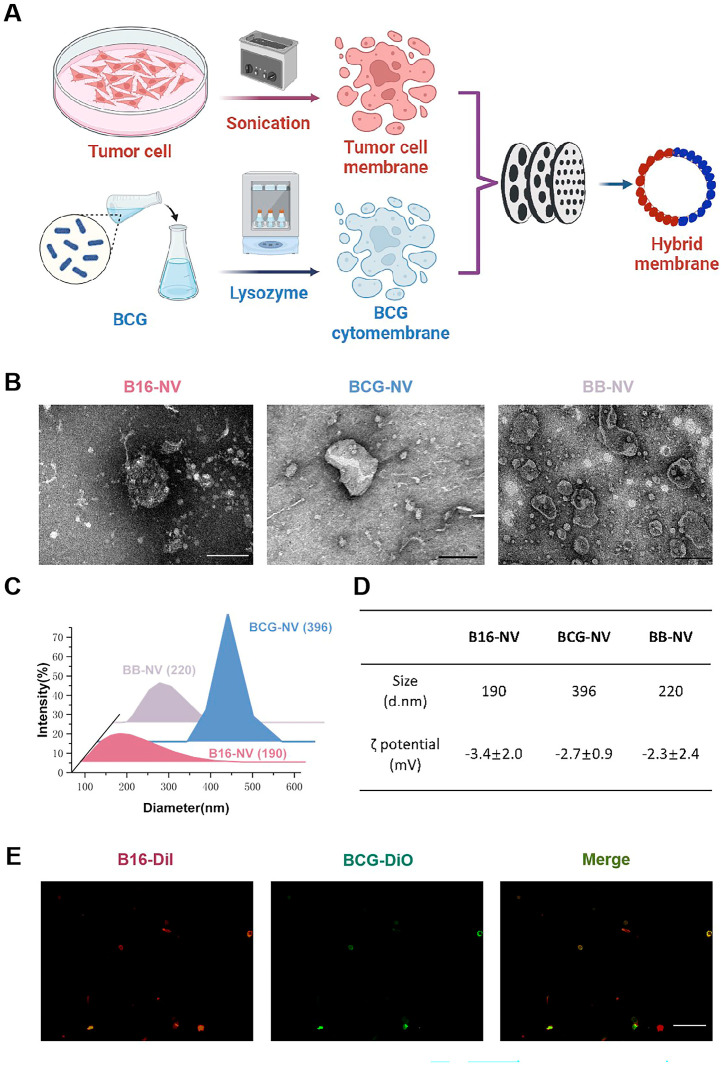
Synthesis and characterization of nanovesicles. (**A**) Schematic diagram illustrating the process of creating BB-NVs. (**B**) TEM images of B16-NVs, BCG-NVs, and BB-NVs. Scale bars = 100 nm. (**C**,**D**) Size distribution and ζ potentials of B16-NVs, BCG-NVs, and BB-NVs; data are presented as means ± SD. (**E**) Characterization of membrane fusion. Tumor cell membranes were labeled by DiI, and BCG cytoplasmic membranes were labeled by DiO. The membrane fusion of the BB-NVs vesicles was visualized under fluorescence microscopy. Scale bars = 1 µm.

All three kinds of nanovesicles were created by extracting the membranes and then creating vesicles of the same size by liposome extruder. The process of extracting melanoma cell membranes involved ultrasonically breaking the cell structure and using gradient centrifugation to eliminate the nuclear substance. BCG cytoplasmic membranes were isolated by destroying the cell wall with lysozyme and gradient centrifugation. The construction of B16-BCG double membrane fusion vesicles was performed by ultrasonically mixing melanoma cell membranes and BCG cytoplasmic membranes before using a liposome extruder (Figure 1A).

Transmission electron microscopy (TEM) images showed that all three nanovesicles had similar morphology and size (Figure 1B). Dynamic light scattering (DLS) analysis was used to measure their average hydrodynamic diameter. It was discovered that BCG-NVs had larger hydrated particle sizes, roughly 396 nm, compared to BCG-NVs and B16-NVs, which had particle sizes of 190 nm and 220 nm, respectively (Figure 1C,D). The disparity between the DLS and TEM particle sizes may be due to the hydrated shell layer formed on the surface of the vesicles when dispersed in an aqueous solution, which leads to a larger particle size [22].

Then, the membrane fusion of BB-NVs was characterized by different cell membrane dyes. B16-NVs were dyed with DiI (red fluorescence) and BCG-NVs with DiO (green fluorescence), while fused BB-NVs were yellow fluorescence, indicating successful fusion of two membrane-based nanovesicles (Figure 1E). To identify the specific components of the nanovesicles, we further detected the markers of B16-NVs, BCG-NVs, and BB-NVs. Ag85B, a specific protein secreted by Mycobacterium tuberculosis, was present on both BCG-NVs and BB-NVs. The melanoma-specific protein, gp100, was detected on B16-NVs and BB-NVs (Figure S1). To evaluate the stability profile of the nanovesicles, we conducted comprehensive stability assessments under various physiological conditions. Initially, we incubated the nanovesicles in three distinct biological media: Dulbecco’s Modified Eagle Medium (DMEM), phosphate-buffered saline (PBS), and fetal bovine serum (FBS) at 4 °C. DLS measurements after 24 h of incubation revealed no significant alterations in particle size distribution across different media, demonstrating excellent media compatibility (Figure S2A). To further characterize the temporal stability, we performed longitudinal monitoring of particle size in PBS at 4 °C over a 96 h period. DLS measurements at 24 h intervals (24, 48, 72, and 96 h) showed consistent particle size distributions, with no statistically significant variations observed throughout the experimental duration (Figure S2B). These findings demonstrate that the nanovesicles possess remarkable stability across different biological media and maintain their structural characteristics over extended periods, suggesting their potential suitability for various biomedical applications requiring prolonged circulation times. All these findings suggested that the dual membrane-based nanovesicles (BB-NVs) exhibit excellent stability and compatibility across different biological media, maintaining consistent particle size over a 96 h period in PBS at 4 °C, indicating their potential for biomedical applications requiring stable and prolonged circulation times.

### 3.2. BB-NVs Increase Tumor Antigen Uptake and Activate Bone Marrow-Derived Dendritic Cells (BMDCs) by Co-Delivery of Antigen and Adjuvant

**Figure 2:**
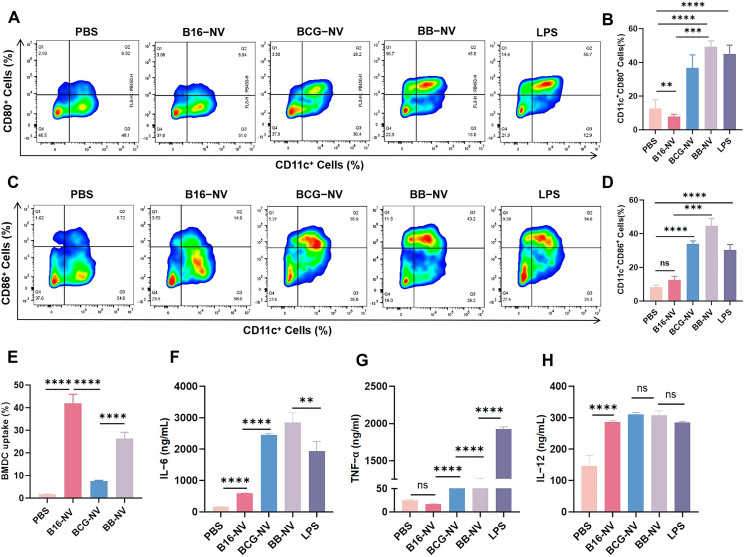
The impact of membrane-fused nanovesicles on DCs in vitro. (**A**) CD11c^+^CD80^+^ DCs as seen in representative flow cytometry images following a 24 h in vitro co-incubation with B16-NVs, BCG-NVs, BB-NVs, and LPS (1 μg/mL). (**B**) The calculated percentage of CD11c^+^CD80^+^ DCs. (**C**) CD11c^+^CD86^+^ DCs as seen in representative flow cytometry images following a 24 h in vitro co-incubation with B16-NVs, BCG-NVs, BB-NVs, and LPS (1 μg/mL). (**D**) The calculated percentage of CD11c^+^CD86^+^ DCs. (**E**) Flow cytometry was used to measure the cellular absorption of FITC-loaded nanovesicles following a 4 h incubation with BMDCs. (**F**) Concentrations of IL-6 (**G**) TNF-α and (**H**) IL-12 in BMDCs supernatants during a 24 h nanovesicles incubation period. (** *p* < 0.01, *** *p* < 0.001, **** *p* < 0.0001, ns stood for *p* > 0.05).

The BCG cytoplasmic membrane is composed of a plasma membrane surrounded by lipid-attached peptidoglycan (PG) and arabinogalactan (AG), which are agonists of TLR2 and TLR4 and can be used as an adjuvant for tumor vaccines to enhance the maturation and migration of DCs in the tumor microenvironment. To assess the immunostimulatory capacity of the material on BMDCs, we conducted a comprehensive flow cytometric analysis. In this study, CD11c was utilized as the specific surface marker for dendritic cell identification, while CD80 and CD86 served as well-established maturation markers for activated dendritic cells. Flow cytometry analysis revealed significant differences in dendritic cell (DC) activation profiles across experimental groups, as measured by CD80^+^CD11c^+^ expression. Compared to the PBS control group, both B16-NVs and BCG-NVs demonstrated substantial DC maturation, with CD80^+^CD11c^+^ DC populations of 8.94% and 28.2%, respectively. The BB-NVs group showed the most robust DC activation among treatment groups, achieving a 45.0% CD80^+^CD11c^+^ DC population. While the LPS positive control group maintained the highest overall DC activation rate at 50.7%, BB-NVs emerged as the most effective treatment condition, demonstrating comparable immunostimulatory capacity to the gold standard LPS control (Figure 2A,B). The CD86^+^CD11c^+^ cell population demonstrated a comparable activation trend to the CD80^+^CD11c^+^ subset. The PBS control and B16-NVs groups showed limited DC activation, with CD86^+^CD11c^+^ percentages of 6.72% and 14.9%, respectively. In contrast, the BCG-NVs treatment induced moderate DC maturation, achieving a 36.9% activation rate. Notably, the BB-NVs group exhibited superior immunostimulatory effects, reaching a 43.2% CD86^+^CD11c^+^ population—surpassing even the LPS positive control group (34.0%) (Figure 2C,D). These data contributed to the hypothesis that co-delivery of antigen and adjuvant can stimulate the immune system in a synergistic manner and may activate APCs more thoroughly than individual antigen and adjuvant nanovesicles.

In addition to the co-delivery of antigen and adjuvant, the effective uptake of the nanovesicles by the APCs is another important factor for immune activation. To investigate whether there was a difference in the uptake of B16-NVs, BCG-NVs, and BB-NVs by DCs, we loaded the vesicles with fluorescent dye FITC. After a 24 h co-incubation period, flow cytometry was used to measure the fluorescence intensity of the vesicle’s uptake by mature BMDCs. Group B16-NVs demonstrated the highest cellular uptake capacity, with 41.8% of DCs showing positive internalization. Notably, group BB-NVs exhibited substantially greater uptake efficiency (26.4%) compared to group BCG-NVs (7.55%), representing a 3.5-fold increase (Figure 2E). It can also be seen from the results that the uptake rate of DCs gradually increased with the increase in tumor cell membrane content. The faster absorption by DCs of the B16-NVs group may be attributed to more appropriate size and antigens on tumor cells’ membrane surfaces.

Then, the anti-tumor cytokine produced by the activated BMDCs was measured. For IL-6, there was a marked increase in concentration from the PBS group (163.0 ng/mL) through B16-NVs (590.8 ng/mL) and BCG-NVs (2444.8 ng/mL), with BB-NVs showing the highest level (2847.5 ng/mL), surpassing even the LPS group (1932.6 ng/mL). The TNF-α concentration showed a significant rise from PBS to BB-NVs (485.4 ng/mL) and BCG-NVs (1189.2 ng/mL), with LPS exhibiting the peak level (1922.4 ng/mL). For IL-12, B16-NVs (286.4 ng/mL) demonstrated a substantial increase from PBS (146.6 ng/mL), and both BCG-NV (311.3 ng/mL) and BB-NV (308.7 ng/mL) maintained high levels without significant differences between them or from LPS (284.7 ng/mL) (Figure 2F–H). The results above show that this membrane-fused antigen–adjuvant co-delivery platform efficiently activates specific anti-tumor immunity by promoting immune cell activation and cytokine secretion.

### 3.3. BB-NVs Vaccination Inhibits Tumor Progression in Murine B16 Tumor Model

**Figure 3:**
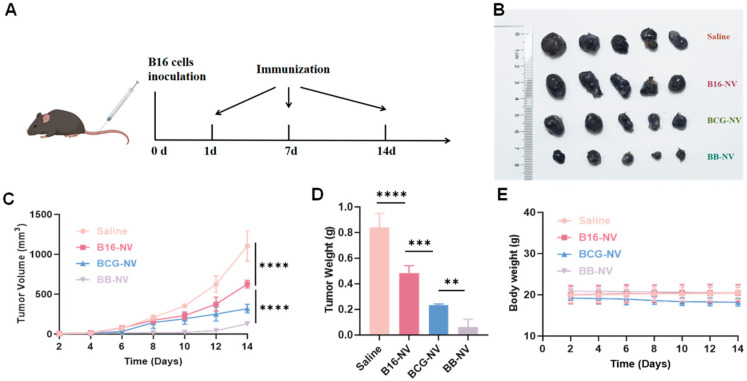
Vaccination with BB-NVs induces tumor regression in the tumor-bearing mouse model. (**A**) Scheme showing the design of the animal experiment. (**B**) The photo of tumor tissues harvested in each group. (**C**) The calculated tumor growth curves. Tumor weight (**D**) and body weight (**E**) of group saline, B16-NVs, BCG-NVs, and BB-NVs. (** *p* < 0.01, *** *p* < 0.001, **** *p* < 0.0001, ns stood for *p* > 0.05.)

To evaluate the tumor suppressive ability, female C57BL/6j mice were subcutaneously inoculated with 1 × 10^5^ murine B16-F10 mouse melanoma cells. Immunotherapy was administered at 1, 7, and 14 days after implantation, and mice were euthanized 24 h after the last immunotherapy (Figure 3A). The monitoring of tumor volume indicated that B16-NVs, BCG-NVs, and BB-NVs all have an anti-tumor effect compared with saline. Among them, BB-NVs had the best tumor-inhibiting effect. Nevertheless, we also discovered that immunotherapy was only able to impede the growth of tumors rather than eradicate them (Figure 3B–D).

Among the mice in the BB-NVs group, we noticed an interesting phenomenon. The local skin alterations occurred at the tumor implantation site in part of animals with the smallest tumors. These mice showed reddish skin and local capillary dilatation at the location of implantation (Figure S3). We postulated that it might be caused by the activation of immune cells in the tumor microenvironment by BB-NVs, which would then trigger an immunological response on the skin [23].

**Figure 4:**
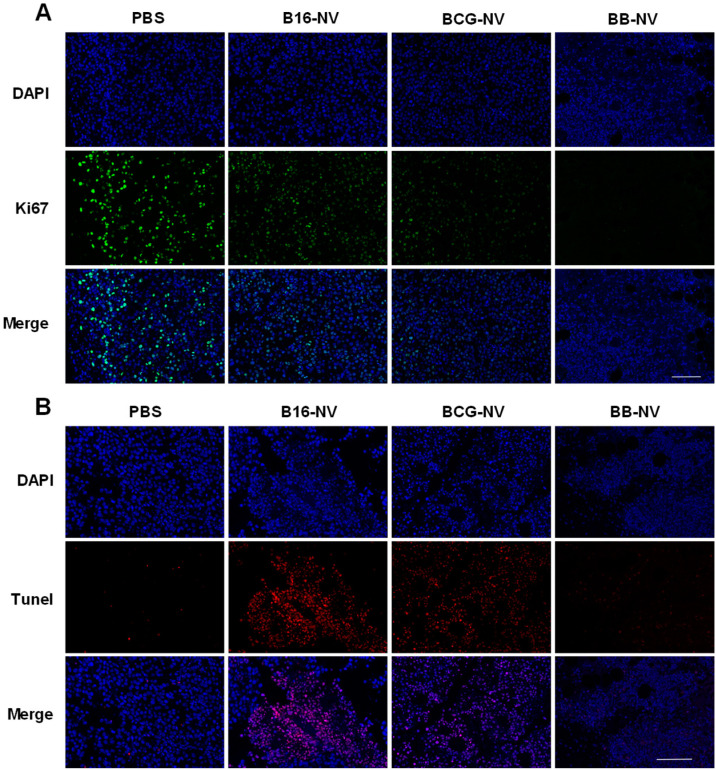
Immunofluorescence staining of mouse tumor tissues. (**A**) Ki67 staining in the tumor tissues of mice that received different treatments (Original magnification, 20×; scale bar = 100 mm). (**B**) Tunel staining in the tumor tissues of mice that received different treatments (Original magnification, 20×; scale bar = 100 μm).

To examine cell proliferation and apoptosis in tumor tissue, we stained excised tumor tissues with Ki67 and Tunel immunofluorescence labeling. It was shown that there was a progressive decline of cell proliferation in the B16-NVs, BCG-NVs, and BB-NVs groups (Figure 4A). The saline control group exhibited the highest proliferation index, with approximately 9.3% Ki67-positive cells. Treatment with B16-NVs resulted in a significant reduction in proliferating cells to 6.53%. The BCG-NVs group demonstrated further suppression of cellular proliferation, maintaining only 1.46% Ki67-positive cells. Most notably, BB-NVs treatment showed the most potent anti-proliferative effect, with Ki67-positive cells reduced to 1.01% (Figure S4A). It is worth noting that the apoptotic trend of the BB-NVs (1.69%) was not evident in Tunel immunofluorescence labeling, whereas the B16-NVs (2.92%) and BCG-NVs (8.0%) groups showed a higher degree of apoptosis (Figure 4B and Figure S4B). We inferred that the reason for this might be that the tumor size in the BB-NVs group was smaller, and the degree of tumor necrosis was not obvious.

### 3.4. BB-NVs Activate Immune Cells and Promote Anti-Tumor Cytokine Secretion in Mice

**Figure 5:**
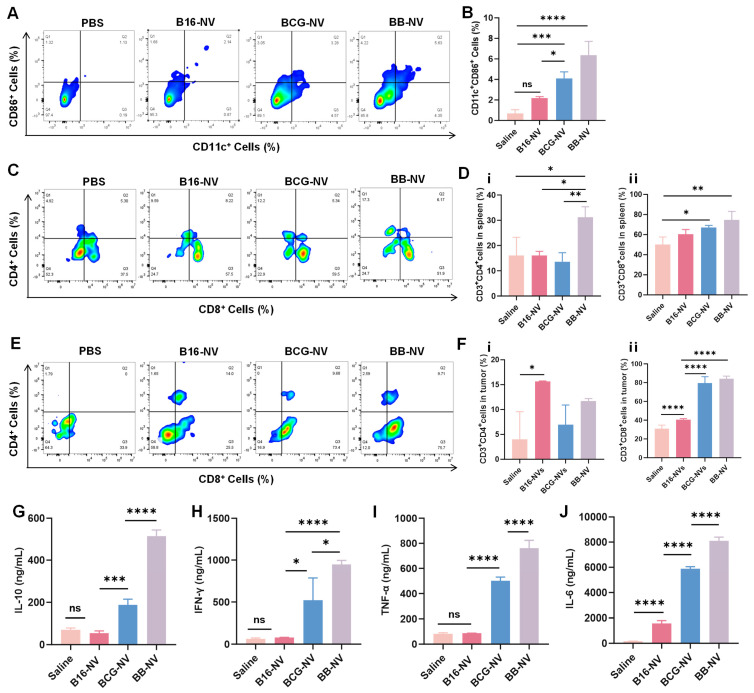
In vivo, BB-NVs stimulate DCs maturation in draining lymph node and T cell activation in the spleen. (**A**) CD11c^+^CD86^+^ DCs from mouse inguinal lymph nodes were detected by flow cytometry. (**B**) The percentage of CD11c^+^CD86^+^ DCs in mice’s inguinal lymph nodes after immunotherapy. (**C**) The spleens of the mice were harvested after three immunotherapy treatments to detect CD3^+^CD4^+^ and CD3^+^CD8^+^ T cells. (**D**) The percentage of CD3^+^CD4^+^ (i) and CD3^+^CD8^+^ T cells (ii) in mice’s spleens after immunotherapy. (**E**) CD3^+^CD4^+^ and CD3^+^CD8^+^ T cells were detected in tumor tissues. Representative flow cytometry images were shown. (**F**) The percentage of CD3^+^CD4^+^ (i) and CD3^+^CD8^+^ T cells (ii) in mice’s tumor tissue after immunotherapy. (**G**–**J**) Concentrations of anti-tumor cytokines in mice blood serum after immunotherapy. (* *p* < 0.05, ** *p* < 0.01, *** *p* < 0.001, **** *p* < 0.0001, ns stood for *p* > 0.05).

To verify the anti-tumor effect of the nanovesicles in animal models, the BMDCs maturation and T cell activation of B16-NVs, BCG-NVs, and BB-NVs groups were assessed in vivo. The same procedure of immunotherapy was conducted as in Figure 3A. The tumor tissue, spleen, and inguinal lymph nodes of tumor-bearing mice were removed for immunological experiment analysis. As shown in Figure 5A,B, the expression of stimulatory markers (CD86) on DCs in mice’s inguinal lymph nodes was significantly higher than in those treated with BB-NVs and BCG-NVs nanovesicles. The highest expression was found in the BB-NVs group (2.18%, 4.10%, and 6.35% for CD86 in the B16-NVs, BCG-NVs, and BB-NVs groups, respectively).

Moreover, the proportion of CD3^+^CD4^+^ and CD3^+^CD8^+^ T cells in the spleens was analyzed by flow cytometry. It was demonstrated that, similar to the results of DCs maturation, BB-NVs also exhibited the highest proportion of CD3^+^CD4^+^ and CD3^+^CD8^+^ T cells in mouse spleen (Figure 5C,D). The T cells were also detected in tumor tissues. As is shown in Figure 5E,F, the B16-NVs, BCG-NVs, and BB-NVs groups showed a trend of progressively higher mature T cell ratios. Among them, the CD3^+^CD8^+^ T cell maturation rate of the BB-NVs group was 84.03%, which was much higher than that of the B16-NVs (40.5%) and the saline group (30.89%). It was demonstrated that adjuvant antigen-integrated membrane-fused nanovesicles could effectively activate APCs and promote the further activation of T cells.

To further investigate the activation of anti-tumor immunity by nanovesicles in vivo, we further examined the secretion of anti-tumor cytokines in the serum of mice (Figure 5G–J). Consistent with in vitro experiments, the B16-NVs group exhibited limited immune activation capacity in vivo. Only IL-6 revealed a propensity to rise, and there was no statistically significant difference observed between the B16-NVs and untreated groups for IFN-γ, IL-10, or TNF-α. BCG-NVs demonstrated distinct immunological activation than the other two groups, as seen by a noteworthy increase in cytokines. The findings demonstrated that BB-NVs retained their capacity to activate the immune system in vivo, hence facilitating immune cell maturation and augmenting the release of anti-tumor cytokines.

### 3.5. Biosafety Assessment of B16-NVs, BCG-NVs, and BB-NVs

**Figure 6:**
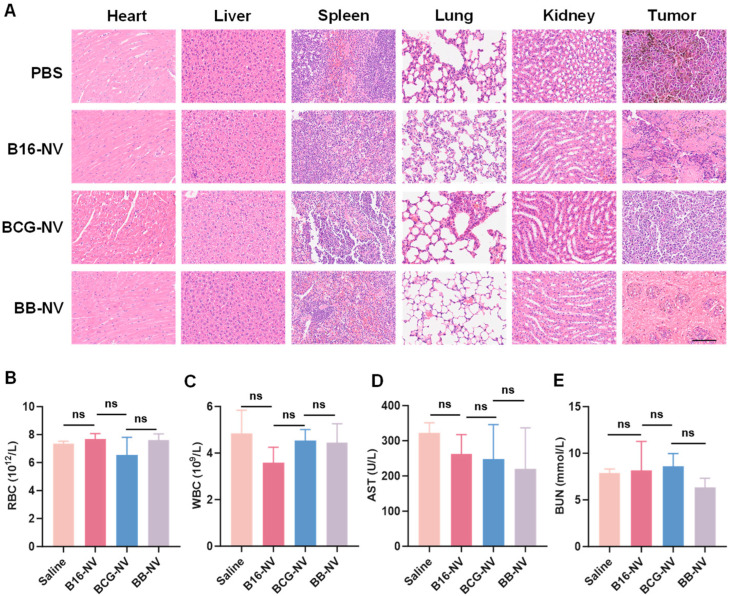
Biological safety assessment of nanovesicles. (**A**) H&E staining of heart, liver, spleen, lung, kidney, and tumor in tumor-bearing mouse model on the 15th day after different treatments (scale bar = 100 μm). (**B**–**E**) Values of routine blood tests (RBC, WBC) and serum biochemistry tests (AST, BUN) of mice in different groups. (ns stood for *p* > 0.05).

The application of the nanomaterials in clinical practice depends heavily on their biosafety. Consequently, to confirm the biocompatibility of materials, the weight change in tumor-bearing mice was monitored. The results showed that none of the mice in the three treatment groups or the control group displayed abnormal weight fluctuations (Figure 3E). Furthermore, according to H&E staining results, no discernible damage can be identified in the groups’ primary organs removed on day 14 (Figure 6A).

We also examined the complete blood count and the liver and kidney functions of the mice in four groups. The results showed that the levels of leukocytes (LYM), red blood cells (RBC), platelets (PLT), blood urea nitrogen (BUN), aspartate aminotransferase (AST), alanine aminotransferase (ALT), and BUN stayed within normal limits. The result demonstrated that B16-NVs, BCG-NVs, and BB-NVs did not induce systemic inflammatory response or impact liver or kidney function in mice (Figure 6B–E and Figure S5). Consequently, the biosafety profile of the double-membrane-fused nanovesicles was satisfactory.

## 4. Discussion

In recent years, autologous tumor cell vaccines have been attempted in clinical trials for metastatic melanoma and solid tumor [24]. However, efficacy was limited due to weak immunogenicity. To improve the efficiency of tumor vaccines, the selection of tumor antigens, as well as the piggybacking of adjuvants, is crucial. However, different release times or sites of antigen and adjuvant may reduce the drug delivery efficiency [25,26]. How to achieve simultaneous delivery of antigen and adjuvant at the same time and space is the key point to improve the effectiveness of tumor vaccines [27]. In this study, we prepared antigen–adjuvant-integrated nanovesicles by membrane fusion. The tumor cell membranes were used as a source of tumor antigens, and the cytoplasmic membranes acted as an immune adjuvant to activate immune cells. (Scheme 1)

Cell membrane-derived vesicles are nano-sized vesicles with a membrane structure that are secreted or artificially synthesized by cells. Studies have shown that extracellular vesicles carry nucleic acids, lipids, and proteins that are involved in intercellular communication and participate in an important role in tumor development [28]. However, in recent years, it has been found that extracellular vesicles are also promising delivery platforms for tumor immunotherapy [11]. Among them, the homologous targeting of extracellular vesicles can enable vesicles to be enriched at the target cell site for the targeted delivery of drugs or nanomaterials. In addition, tumor-derived vesicles or bacterial-derived vesicles have the ability to activate APCs. However, the function of a single source of vesicles may be limited, and to ameliorate this drawback, researchers have merged the advantages of different species of vesicles through synthetic membrane-fused nanovesicles [29].

A drug repurposing strategy was used for the bacterial selection in this study. The BCG vaccine, an attenuated live strain of *Mycobacterium bovis*, is one of the most widely used vaccines in the world and is now widely used in the treatment of prostate cancer, where intravesical injection of BCG has been shown to reduce the recurrence and progression of non-muscle invasive bladder cancer [30]. BCG instillation intravenously can stimulate the immune system effectively by increasing long-term immunological memory in the form of antigen-specific B and T cells. BCG has also been shown to establish innate immunological memory or “trained immunity” in innate immune cells such as monocytes and NK cells [31,32]. Trained immunity involves germ line-encoded pattern recognition receptors (PRRs), such as TLRs, NOD2, and Dectin 2 [33,34,35], to recognize conserved pathogen- and danger-associated epitopes or molecular patterns (PAMPs and DAMPs) [36].

In recent years, BCG has also been used in melanoma immunotherapy. The obstacles that have prevented BCG from being utilized clinically in melanoma cases are the risks of TB dissemination and its systemic immune response. Based on these results, we modified the BCG vaccine’s delivery form. We separated the immunogenic cytoplasmic membranes and combined them with melanoma cell membranes by a liposome extruder to create membrane-fused nanovesicles of the right size and homogeneity.

In this study, we prepared three types of nanovesicles, namely, tumor cell membrane nanovesicles B16-NVs, BCG-NVs with BCG cytoplasmic membrane nanovesicles, and nanovesicles with tumor cell bacteria double membrane fusion. The sizes of these three vesicles are more easily absorbed by APCs. According to our research, they could boost the uptake by DCs. Furthermore, there was an increased capacity for DCs activation and a higher degree of anti-tumor cytokine secretion using the antigen–adjuvant co-delivery method.

In addition, the membrane-fused nanovesicles also showed excellent immune activation, as well as anti-tumor ability in experiments in vivo. In vivo studies showed that treatment with BB-NVs increased the proportion of CD3^+^CD4^+^ and CD3^+^CD8^+^ cells in tumor tissue and spleen and significantly increased the expression of anti-tumor cytokines TNF-α, IL-6, IL-10, and IFN-γ. Moreover, the biosafety profile of all three vesicles was satisfactory.

As a result, we developed an integrated membrane fusion antigen–adjuvant concept that has demonstrated satisfactory immunotherapy efficacy for melanoma. This membrane-fused nanovesicle serves as a versatile delivery platform and is an effective biomaterial for immunotherapy. This nano-vaccine acts as a good piggyback platform, in addition to presenting tumor antigens to activate APCs. By piggybacking drugs or imaging materials inside the vesicles, we may develop multimodal therapeutic strategies for melanoma.

## 5. Conclusions

In this study, we have successfully created nanovesicles that combine the cytoplasmic membrane of bacteria with the tumor cell membrane. The vesicles have the potential to enhance vesicle uptake by DCs and immune cell maturation greatly. The dual-membrane fusion nanovesicles in a B16 mouse melanoma model activated anti-tumor immunity and increased immune cell infiltration in the tumor microenvironment by inducing the maturation of T cells and DCs in the draining lymph nodes. They demonstrated effective anti-tumor efficacy and a strong biosafety profile. These results offer a workable plan for improving the effectiveness of clinical autologous cancer vaccines.

## Data Availability

Data are contained within the article.

## Supplementary Materials

The following supporting information can be downloaded at: https://www.mdpi.com/article/10.3390/pharmaceutics17040507/s1, Figure S1: The western blot analysis of gp100 and Ag85b in BB-NVs, BCG-NVs and B16-NVs. Figure S2: Detection of the stability of nanovesicles. (A) Changes in particle size of B16-NVs, BCG-NVs, and BB-NVs in different solvents. (B) Changes in particle size of B16-NVs, BCG-NVs, and BB-NVs at different time points. Figure S3: Following immunotherapy, mice in the BB-NVs group developed white hard nodules beneath their skin or red plaques. Figure S4: (A) The mean and standard deviation of Ki67 staining in each group. (B) The mean and standard deviation of Tunel staining in each group. Figure S5: Values of routine blood tests (A) Hb, (B) PLT, (C) LYM, and (D) MCH and serum biochemistry tests (E) ALT of mice in different groups.

## Abbreviations

APC	Antigen-Presenting Cells
BCG	Bacillus Calmette–Guérin
DMEM	Dulbecco’s Modified Eagle Medium
DCs	Dendritic Cells
FBS	Fetal Bovine Serum
PBS	Phosphate-Buffered Saline
TEM	Transmission Electron Microscopy
DLS	Dynamic Light Scattering
ELISA	Enzyme-Linked Immunosorbent Assay
FITC	Fluoresce-Activated Cell Sorting
H&E	Hematoxylin and Eosin Staining
SEM	Scanning Electron Microscopy
°C	Celsius
