# Supplementary material for: A Bionic “Trojan Horse”-like Nanovesicle Delivery System Hybridized with BCG Cytoplasmic Membrane and Melanoma Cell Membrane for Cancer Immunotherapy
Source: Pharmaceutics. 2025 Apr 11;17(4):507. doi: 10.3390/pharmaceutics17040507 (PMC12030220; doi:10.3390/pharmaceutics17040507)
Supplement: Supplementary file 1 [file pharmaceutics-17-00507-s001.zip › pharmaceutics-3490974-supplementary.pdf]

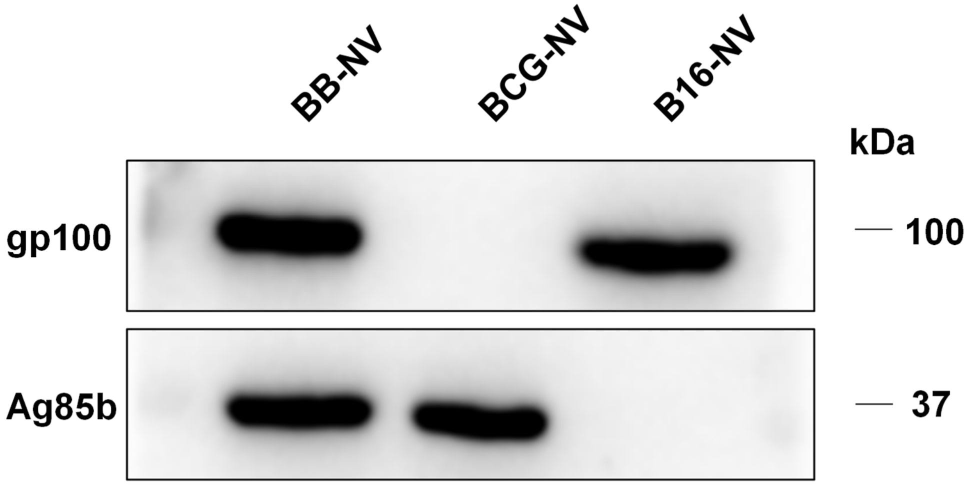

Figure S1. The western blot analysis of gp100 and Ag85b in BB-NVs, BCG-NVs and B16-NVs.

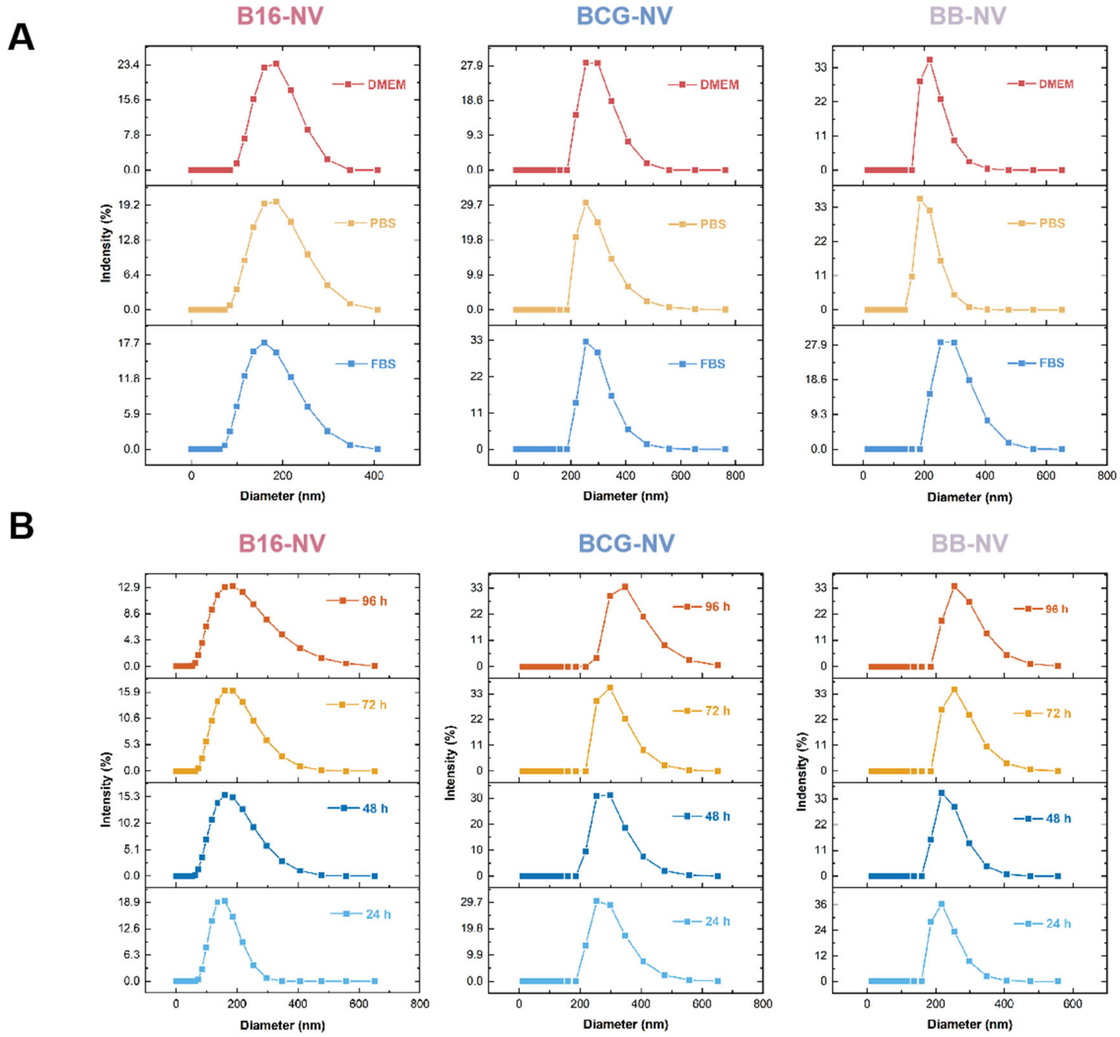

Figure S2. Detection of the stability of nanovesicles. (A) Changes in particle size of B16-NVs, BCG-NVs, and BB-NVs in different solvents. (B) Changes in particle size of B16-NVs, BCG-NVs, and BB-NVs at different time points.

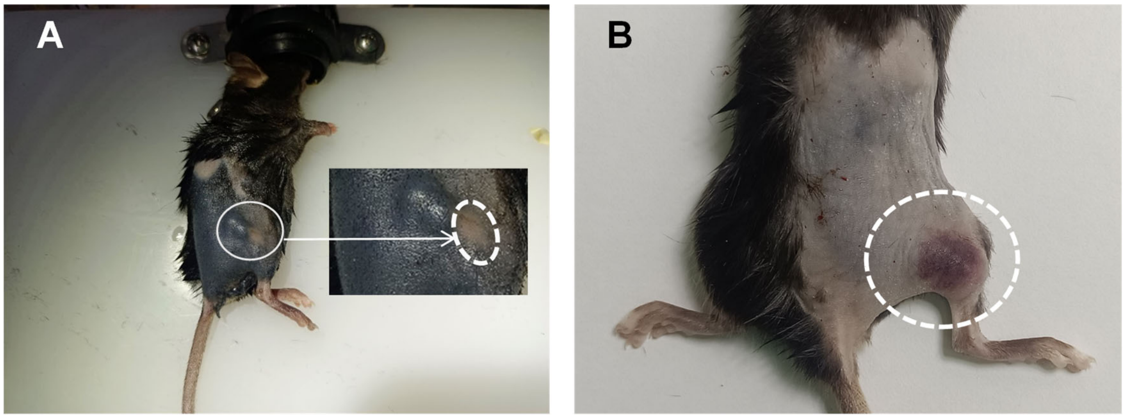

Figure S3. Following immunotherapy, mice in the BB-NVs group developed white hard nodules beneath their skin or red plaques.

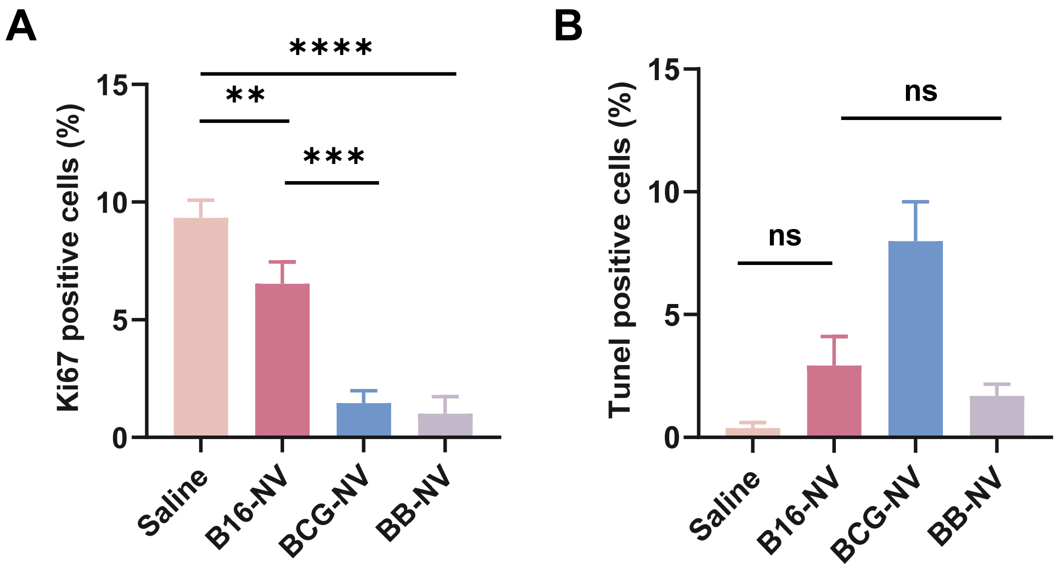

Figure S4. Immunofluorescence of mouse tumor tissues.(**A**) The mean and standard deviation of Ki67 staining in each group. (**B**) The mean and standard deviation of Tunel staining in each group.

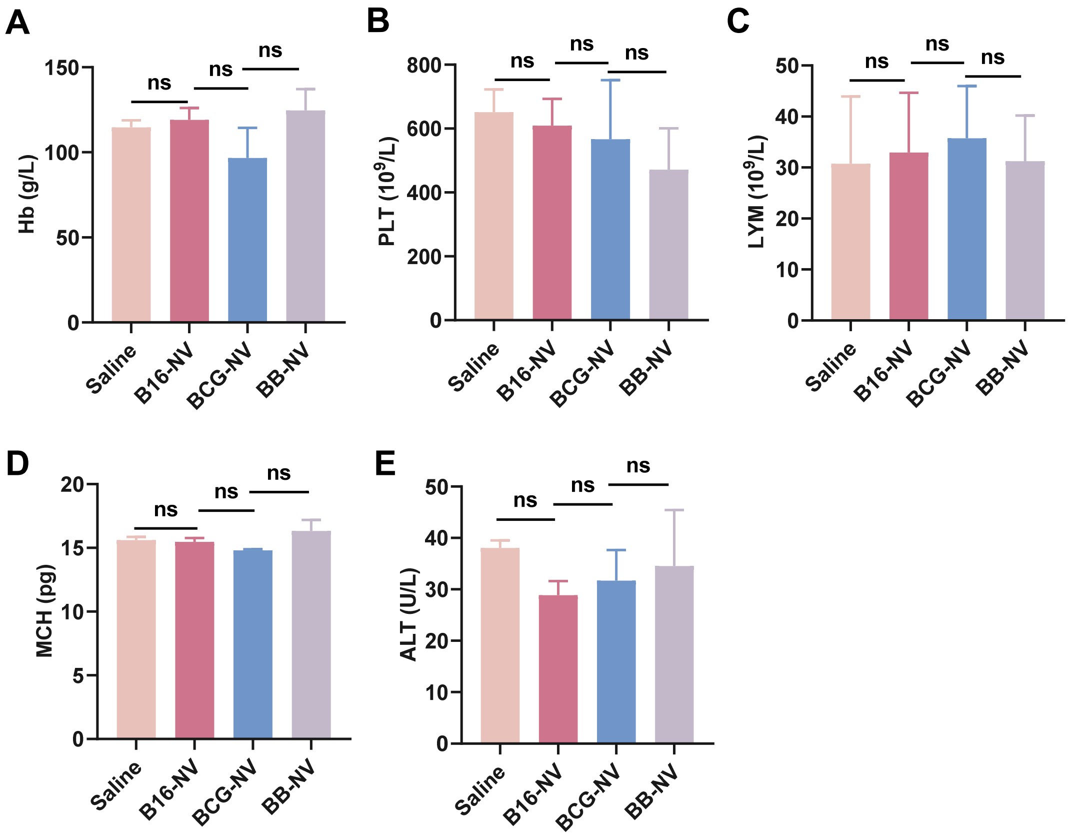

Figure S5. Values of routine blood tests (Hb, PLT, LYM, and MCH) and serum biochemistry tests (ALT) of mice in different groups.
